# Grasp Detection from Human ECoG during Natural Reach-to-Grasp Movements

**DOI:** 10.1371/journal.pone.0054658

**Published:** 2013-01-24

**Authors:** Tobias Pistohl, Thomas Sebastian Benedikt Schmidt, Tonio Ball, Andreas Schulze-Bonhage, Ad Aertsen, Carsten Mehring

**Affiliations:** 1 Faculty of Biology, University of Freiburg, Freiburg, Germany; 2 Bernstein Center Freiburg, University of Freiburg, Freiburg, Germany; 3 Epilepsy Center, University Hospital, University of Freiburg, Freiburg, Germany; 4 Department of Bioengineering and Department of Electrical and Electronic Engineering, Imperial College London, London, United Kingdom; 5 Institute of Neuroscience, Newcastle University, Newcastle Upon Tyne, United Kingdom; Tel Aviv University, Israel

## Abstract

Various movement parameters of grasping movements, like velocity or type of the grasp, have been successfully decoded from neural activity. However, the question of movement event detection from brain activity, that is, decoding the time at which an event occurred (e.g. movement onset), has been addressed less often. Yet, this may be a topic of key importance, as a brain-machine interface (BMI) that controls a grasping prosthesis could be realized by detecting the time of grasp, together with an optional decoding of which type of grasp to apply. We, therefore, studied the detection of time of grasps from human ECoG recordings during a sequence of natural and continuous reach-to-grasp movements. Using signals recorded from the motor cortex, a detector based on regularized linear discriminant analysis was able to retrieve the time-point of grasp with high reliability and only few false detections. Best performance was achieved using a combination of signal components from time and frequency domains. Sensitivity, measured by the amount of correct detections, and specificity, represented by the amount of false detections, depended strongly on the imposed restrictions on temporal precision of detection and on the delay between event detection and the time the event occurred. Including neural data from after the event into the decoding analysis, slightly increased accuracy, however, reasonable performance could also be obtained when grasping events were detected 125 ms in advance. In summary, our results provide a good basis for using detection of grasping movements from ECoG to control a grasping prosthesis.

## Introduction

Brain-machine interfaces (BMI) aim to restore movement and communication abilities of paralysed patients. To this end, movement intentions are read out from brain activity and translated into actions of external actuators. For such devices, movement decoding from neural activity can be carried out continuously over time, for example by continuously decoding the intended state of the effector (e.g., position and velocity of hand and arm joints) at each point in time and translating the decoded state into corresponding movements of a prosthesis. Such a decoding scheme was applied e.g., by Velliste and colleagues [1] to let monkeys continuously control the opening and closing of a gripper. However, to implement different grasp modes, the number of involved hand joints increases, requiring simultaneous and continuous control of a high number of degrees of freedom. An alternative BMI control scheme is to decode a discrete set of movement classes, e.g. different kinds of natural grasps. This, however, requires the additional detection of the time of the movement event, that is, the time at which the grasp should be applied.

While classification of different movement types has been extensively studied in primates and humans [2], the question of movement event detection from neuronal activity was addressed less often. Some previous studies on event detection dealt with the detection of the onset of reaching movements [3], [4] or the onset of hand/wrist extensions [5], [6], using a variety of detection methods, signal features and recording techniques: Hwang and Andersen [3] detected the onset of monkeys’ reaching movements from the difference of the temporal derivatives of 20–40 Hz and 0–10 Hz power of the local-field potential, using a thresholding mechanism. Studies on humans used different classification algorithms on spectral features of the EEG to detect hand extensions [5], [6]. The frequency of the used spectral frequencies varied widely: Awwad Shiekh Hasan and Gan [6] modelled spectral EEG features in the range of 8–45 Hz with a mixture of Gaussians, whereas Bashashati and colleagues [5] used spectral power in bands between 1 and 25 Hz for linear discriminant analysis. The latter also tested a nearest neighbour classifier on low-pass filtered EEG. Another approach was applied by Levine and colleagues [4] who based detection of various movements and vocalizations on the cross-correlation of recordings of the human electrocorticogram (ECoG) with average evoked potentials for the various events.

Movement events of interest may also be embedded within a larger sequence of sub-movements, without pronounced pauses, disqualifying detection of a general onset of movements. For example, this is the case when the time of grasping should be detected during natural, continuous reach-to-grasp movements. So far, little is known about such detection of grasping movements from brain activity.

We created a movement paradigm in which grasping movements are occurring during a sequence of self-paced and largely self-chosen movements. We previously showed that different modes of grasping can be reliably decoded from human ECoG under these conditions [7]. Here, we demonstrate that the time of the grasping movements can also be detected from the same data. We quantify the precision of detection as a function of various parameters and show that reasonable precision can be obtained for real-time applications where movement events need to be predicted before, or detected while they are produced.

## Methods

### Subjects

Three subjects, who will be referred to as S1, S2 and S3, participated in our study. Subjects had a number of ECoG electrodes subdurally implanted for presurgical epilepsy diagnostics. All three subjects were female and 14 to 16 years of age. Information about implantation sites and pathology can be found in table 1. The study was approved by the University Clinic’s ethics committee and was conducted only after subjects and their parents (since subjects were under-age) had given their informed written consent.

**Table 1:** Subject information.

	S1	S2	S3
*age*	14 years	16 years	15 years
*handedness*	right	right	right
*pathology*	right frontal FCD	FCD in right superior frontal gyrus/rightcingulated gyrus	right frontal FCD
*implanted electrodes*	fronto-parietal 8×8 grid; 3 lateral prefrontalstripes (1×6); 1 anterior cingulated depthelectrode (10 contacts); 1 medial fronto-polardepth electrode (10 contacts); all electrodeson the right	fronto-parietal 6×8 grid; 3 interhemisphericstripes (1×4); all electrodes on the left	right fronto-parietal 8×8 grid
*seizure onset zone*	right medial and lateral prefrontal	left interhemispheric	right premotor

All subjects were female and had ECoG electrodes subdurally implanted for pre-neurosurgical diagnosis. FCD: focal cortical dysplasia.

### Experimental Task

**Figure 1:**
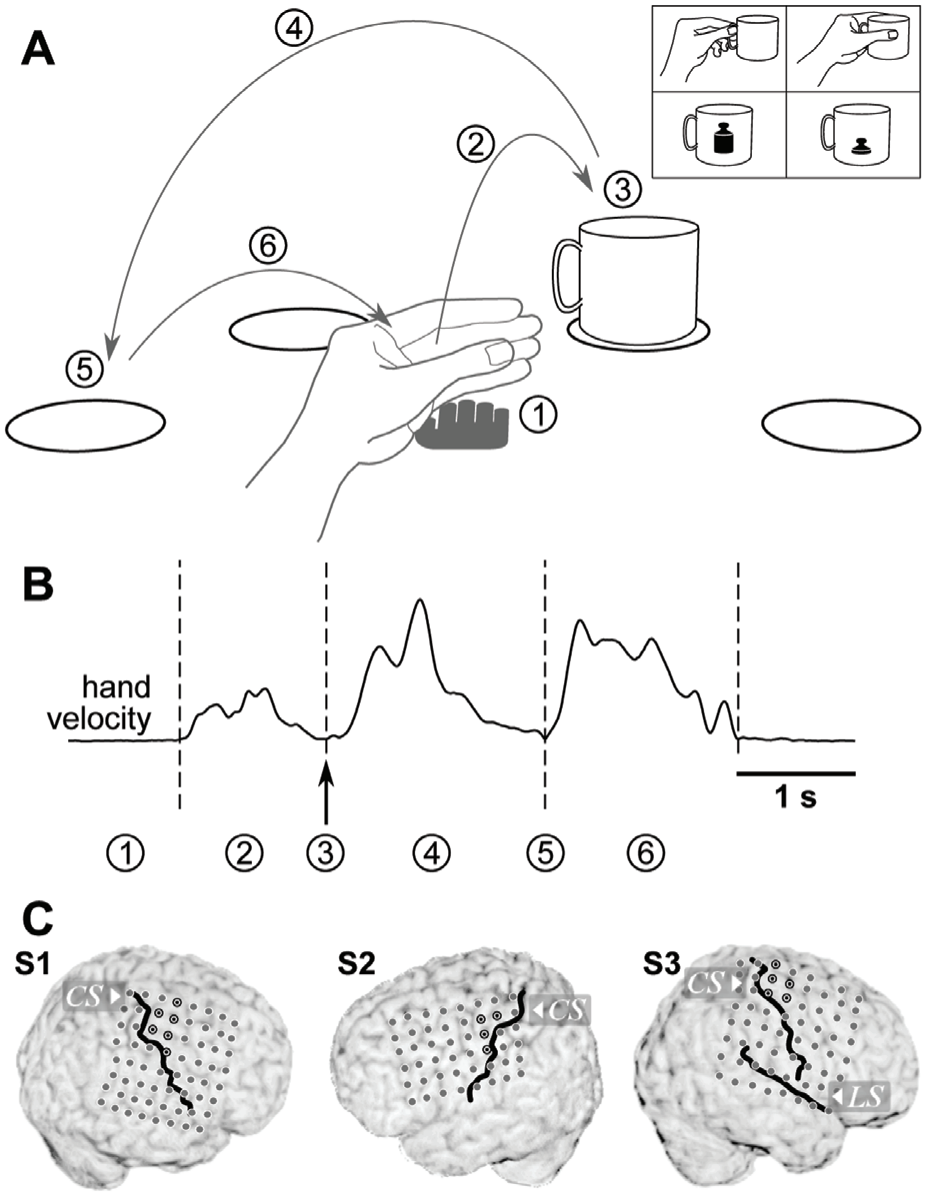
Experimental paradigm. A: Task layout. The subject’s hand was resting palm down (1) on a central spot, marked by the grey hand pictograph. A reaching movement (2) was initiated by the subject (self-paced) and a cup was grasped (3) at one of four marked positions (circles) and carried (4) to one of the remaining three positions (self-chosen). There, the cup was released (5) and the hand returned (6) to the central resting position. Grasps of the object varied with respect to the applied grasp type (precision or whole-hand grip, self-chosen in every trial) and object weight (switched between two cups every 15–16 trials), pictured in the upper right inset. B: Sample profile of hand velocity during one trial. Actions labeled by numbers in (a) are marked at their respective times. C: Electrode implantations in all three subjects. The position of the central sulcus and the lateral sulcus (S3 only) relative to the electrodes are shown by black lines. Electrode contacts of hand-arm motor cortex are marked by a black circle with a black dot in the center.

We created a task, in which grasping movements were part of longer natural reach-to-grasp movements. The movement paradigm is outlined in figure 1a. Reaching movements were self-initiated by the subject by reaching from a marked resting position to a cup, placed at one of four locations, arranged in a semi-circle around the resting position and drawn on a flat table in front of the subject. Without explicit halt, the cup was lifted and carried to one of the other marked positions, where it was released. After that subjects moved their hand back to the resting position. Several factors increased the variability of grasping movements:

The starting position of the cup changed in every trial, being the position it was carried to in the previous trial.

The cup could be grasped in two different ways: with a precision grip at the handle or a whole-hand grip around the cup. Subjects were asked to choose either of the two grasp types with equal probability in each trial.

After each block of 15 to 16 trials, the cup alternated between an empty, light-weight version (68 g) and a heavier version (340 g), with some weights fixed to the bottom of the cup.

These factors ensured a large amount of variability of grasping events, making detection a non-trivial undertaking.

Due to the self-paced nature of the task, the time of the grasping event in each single trial had to be subsequently identified from the behavioural data. ‘Time of grasp’ here relates to the moment in time when the grip was tightened, shortly before lifting the cup. The transitions between movement components were smooth, without pronounced separation. The time of grasp could be defined with a precision estimated to about 60 ms.

For S1 and S3, we used recordings of wrist position, obtained synchronously to the neural data using an ultrasound tracking system (Zebris, Isny, Germany). The time of grasp was marked by a local minimum in hand speed (figure 1b) and by a simultaneous minimum in hand elevation over the table and a turning point in the trajectory parallel to the surface. For S2, the recordings of wrist position could not adequately be synchronized to the neural recordings. We therefore used video recordings of the subject, acquired synchronously and routinely in the course of clinical observation. Similar criteria as for S1 and S3 were applied to define the time of a grasping event, but based on a frame-by-frame video analysis. With video frames recorded every 40 ms, and an ambiguity of about one frame (earlier or later) for the identification of these events, the imprecision of grasping events was estimated to be about 60 ms and, therefore, similar to the temporal precision estimated for event times derived from wrist position recordings.

Table 2 gives an overview over number of grasps and analysed time for each subject.

**Table 2:** Amount of analysed data.

	*number of grasps*	*median inter-grasp time*	*total time analysed*	*non-movement time*
**S1**	303 in 20 blocks	5.2 s	1919 s ( = 32.0 min)	657 s
**S2**	338 in 21 blocks	5.3 s	2119 s ( = 35.3 min)	989 s
**S3**	320 in 20 blocks	4.6 s	1522 s ( = 25.4 min)	401 s

Compound movements, including gripping and carrying a cup, were self-paced, with short resting periods between trials, and between blocks of trials to allow alternating between a light-weight and a heavier cup. Subjects decided for either a precision or a whole-hand grip on a trial-to-trial basis. The total time analysed also included non-movement time between trials.

### Neural Recordings

Subjects were implanted with stainless steel electrodes (Ad-Tech, Racine, Wisconsin, USA) of 4 mm diameter, covered in sheets of silicone, arranged in regular grids with 10 mm inter-electrode distance. Electrode arrays were implanted subdurally over the lateral convexity of subjects’ cortices, partly covering precentral motor cortex. The choice of electrode implantation sites was exclusively based on clinical requirements, unrelated to the experiment.

The electrocorticogram (ECoG) was recorded using a clinical EEG-System (IT-Med, Germany), and sampled at a rate of 256 Hz (S1, S2) or 1024 Hz (S3). A digital video recording (25 Hz frame rate), synchronized to the ECoG, was additionally acquired for all subjects.

Electrical stimulation was performed through the electrode grid. All sites with arm or hand motor responses were, in all subjects, located outside the ictal onset zone.

In each subject, a structural MRI data set with full head coverage was acquired, both before and after electrode implantation. Motor cortices were identified according to anatomical landmarks [8]–[10]. The positions of the central and lateral sulci relative to the electrode positions were determined from the post-implant MRI.

Electrode contacts residing over hand-arm motor cortex were identified by two conditions, which both needed to be met: (i) their precentral anatomical location, and (ii) by hand or arm movements evoked upon electrical stimulation through these electrodes. Figure 1c highlights these locations by a black outline and a dot. As a likely target area for BMI applications, all analyses presented here were performed exclusively on signals recorded from this cortical area.

### Data Processing

#### General treatment of data

Prior to any further processing, data recorded by the clinical EEG system was re-referenced to a common average reference. For each recorded channel, the average voltage over the entire recording time was subtracted to eliminate any possible offset of the signals. To account for systematic differences in amplitudes across channels, which would prevent direct comparison between channels, signals of each channel were also divided by their respective standard deviation over time.

Our main analysis was aimed to detect events within continuous stretches of ECoG data. Some longer pauses between trial blocks were introduced when subjects needed to rest or readjust their position or some other interruption to the experiment occurred. During this time subject behaviour, which may or may not have included instances of grasping movements, could not be accurately controlled or protocolled, precluding evaluation of potential detections. Therefore, we restricted analysis to continuous periods within blocks of trials and some shorter breaks in-between. Periods of data that were temporally separated from grasping events further than 4 times the median inter-grasp interval (about 20 seconds) were excluded from analysis. This procedure further ensured that the proportion of movement to non-movement times was comparable between subjects. The length of data analysed from each subject can be found in table 2, part of which was spent without overt hand movement, in-between trials (last column of table 2).

#### Low-pass filtering

**Figure 2:**
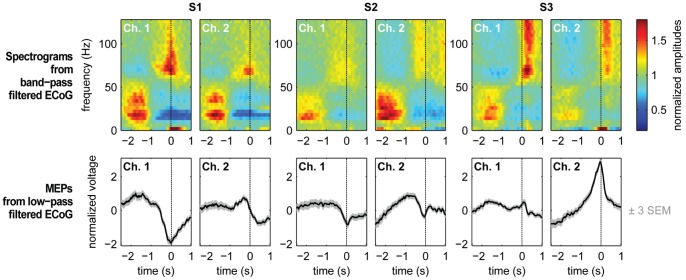
Event-related signals. Signal components of the ECoG recorded from two example electrodes over hand-arm motor cortex of each subject. Top row: Average spectrograms, aligned on the time of grasp. Spectrograms were computed by (causal) band-pass filtering in successive bands of 4 Hz width and subsequent rectification and smoothing with a (causal) Savitzky-Golay filter. Estimated amplitude modulations were normalized by the average amplitude, per frequency bin and channel, over the entire recording. Bottom row: Trial-averaged low-pass filtered ECoG signals (causal Savitzky-Golay filter), aligned on the time of grasp. Gray bands around the black traces of average potentials depict three times standard error of the mean (SEM) in positive and negative direction.

We used a causal version of a 2^nd^ order Savitzky-Golay-filter of 250 ms length to smooth recorded ECoG signals. This corresponds to a low-pass filter with an approximate 3-dB-cutoff frequency at 6.7-Hz. We termed the resulting signal the low-pass filtered component (LFC). Two examples of trial-averaged LFC from each subject, aligned on the time of grasp, are shown in the bottom row of figure 2. Example channels were chosen from the hand-arm motor area, marked in figure 1c.

Low-pass filtered EEG, MEG, ECoG and LFPs have already been used successfully to determine movement directions [11]–[14] and trajectories of continuous hand-arm movements [11], [15], [16].

#### Frequency band amplitudes

In addition to low-pass filtering, we also extracted modulations of amplitudes within consecutive bands of 4 Hz width, from 0 to 128 Hz. This was done by band-pass filtering, rectification of the filtered signal and subsequent low-pass filtering, using the same low-pass filter, described above. We chose this method because it can easily be implemented in a causal way and accurately synchronized to the LFC. For band-pass filtering, we chose a 4^th^ order elliptic digital filter design [17] for its steep roll-off characteristics and because it introduces only small temporal shifts due to phase distortions in the filtering process. The amplitudes of each 4 Hz band were normalized by the average amplitude of this band over the whole recording time. This normalization prevents frequency bands with overall weaker signal power, especially high-frequency bands, from being under-represented when averaging amplitudes over broad frequency ranges [12]. Trial-averaged amplitudes, in time and frequency, are shown in the upper row of figure 2, presenting two exemplary hand-arm motor channels per subject.

Amplitudes of different frequency bands are often used to infer motor behaviour from neural activity recordings, such as LFP, ECoG or EEG e.g., [12]–[15], [18]. If amplitudes are consistently modulated over broader frequency bands, averaging over these bands can significantly improve signal-to-noise ratio, compared to that of narrower frequency bands.

### Event Detection

We distinguished between two classes: ‘event’ (occurrence of a grasp) and ‘non-event’ (no grasp). The feature vector *x* contained signal features extracted from the neural recordings, that is, voltage values or amplitude envelope values of different frequency bands obtained from different ECoG recording sites at a given time, attributed to either event or non-event class (see paragraph ‘Construction of feature vectors’ below for details).

#### Regularized linear discriminant analysis

We employed regularized linear discriminant analysis [19] to decide whether or not an event occurred at a specific time. Starting from Bayes’ theorem, the posterior probability *P*(*C_i_|x*) for class *C_i_*, given observation *x*, is given by
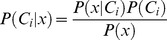



where *P*(*x|C_i_*), called the likelihood, is the conditional probability of *x*, given class *C_i_*, *P*(*C_i_*) the prior probability of class *C_i_*, and *P*(*x*) the prior probability of observation *x.* Prior probabilities *P*(*C_i_*) of all occurring classes can be estimated from training data with known class assignments. Estimation of the conditional probabilities *P*(*x|C_i_*) is based on an approximation of the distribution of observations *x* for each class *C_i_* by a (multi-variate) *N*-dimensional Gaussian distribution. In linear discriminant analysis (LDA), it is, additionally, assumed that all class distributions have the same covariance matrix *Σ* and only differ in their means. Class-dependent means and the common covariance matrix were also estimated from training data. Finally, *P*(*x*) can be computed as 
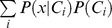
.

Since a maximum likelihood estimate of the covariance *Σ* based on a limited amount of training data can easily lead to overfitting in a high-dimensional feature space, we used regularized LDA. This imposes additional restrictions on the covariance matrix, by interpolating between the maximum likelihood estimate of the covariance matrix *Σ* and the scalar covariance [19]:
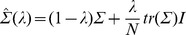



Here, *I* denotes the identity matrix and tr(*Σ*) the trace of *Σ*. The degree of interpolation is specified by the regularization parameter 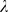, used to obtain the regularized covariance 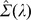. A value of 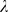 = 0 corresponds to a non-regularized linear discriminant analysis, whereas 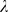 = 1 assumes spherical Gaussian distributions. We used values of 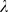 from a set of [0.0001, 0.001, 0.01, 0.1, 0.5, 1], optimized on the respective training sets (see section ‘Evaluation of detections’).

#### Construction of feature vectors

Decoding is based on a neuronal feature vector. The selection of neuronal features can be tailored in several ways. One important choice is which signal components to use. Another question is, at which time related to the decoded property, in our case the grasping event time, features should be extracted from the neuronal signals. Here, we call this latter property the *delay*, which is a free parameter in our analysis. A negative *delay* corresponds to a time prior to the event, a positive *delay* to a time after the event. The choice of the *delay* is constrained by the time at which the detection should be available: If it is sufficient to learn about detection after the actual event, signals occurring after the event might still be taken into account. Post-event signals can be informative, since post-event neural processing and behaviour could still be related to the initial event (e.g., holding a cup is related to previous grasping). If, however, the detection should be known before the event i.e., if a prediction of a future event is required, only negative *delays* should be allowed.

It can also be beneficial to enlarge the feature space by using neuronal features from multiple time points. For simplification, features can be collected at fixed intervals throughout an epoch of exploited signal history, sampled such that most information from the signals is retained. Since all signal components, used here, were low-pass filtered (see above) and, therefore, had most of their power below 8 Hz, we restricted ourselves to 16 time points per second of signal history. For example, when using a signal history of one second, the feature vector for time *t* included 17 samples of each channel, including a sample from *t - delay* and 16 samples from earlier times, with the earliest sample recorded at *t - delay* - 1 s. To use the signals from multiple channels, the feature vectors of all channels were concatenated. The length of a feature vector, using one signal component, thus depends on the length of the signal history 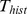 and the number of included ECoG channels, and can be calculated as 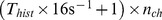. We created such a vector for each analysed time step.

To fit the mean and covariance of the event class to a subset of the available recordings (training set), feature vectors were extracted from the training data at every event time contained therein. For the non-event class training set, however, we did not simply use all remaining time points, as this could lead to several problems: First, the batch of non-event training data would grow exceedingly large, making it computationally costly. Moreover, due to autocorrelations in the signals, neighbouring samples are mutually dependent. This would introduce redundancy and, in addition, make non-event samples close to grasp times very similar to event samples, leading to weaker separation of the two classes, that is, a greater overlap of class distributions. To avoid these issues, we excluded times closer than 300 ms to the next event from the training set and restricted the number of non-event samples to 16 times the number of event samples in the corresponding subset of data. These non-event-samples were gathered from times evenly distributed over the remaining part of the training data. In the test data, used for subsequent event detection, however, no such selection was made.

#### Determination of detected times

**Figure 3:**
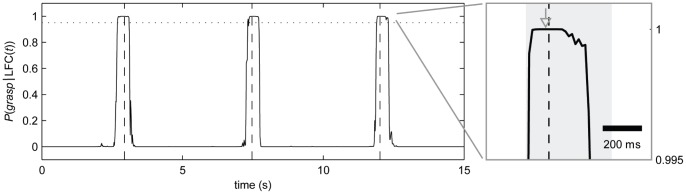
Illustration of the detection process. Example trace of the posterior probability P(grasp|LFC(t)) for a grasping event, given the LFC of the ECoG at time *t*. Vertical dashed lines mark the actual event times. Times with a posterior probability >0.95 (horizontal, dotted line) are potential detections. The corresponding time interval around one detection is shown as a grey shaded area in the enlarged section on the right. Final detection time is marked by a grey arrow. See Methods, section ‘Event detection’, for details of the detection algorithm.

We calculated the posterior probability *P*(*event|x*) to observe an event, given the measured signals, every 15.625 ms i.e., 64 times per second. *P*(*event|x*) larger than 0.5 signified that an event should be considered more probable than no event. However, since the signal components used in the feature vector *x* were autocorrelated on short time scales, a whole set of time points around the actual time of the event yielded high posterior probabilities (figure 3). The threshold required to trigger a detection was set to *P*(*event|x*)>0.95. Peaks in the posterior probability of the event class were usually quite distinct from periods with no event, and quite broad in time, as exemplified in figure 3. In theory, the most likely one in a set of consecutive time points with high *P*(*event|x*) i.e., the peak time of the posterior probability, could be used. However, such maxima can only be registered retrospectively, when data after the peak have already been recorded and analysed. To develop an approximation of this strategy which is still compatible with real-time usage, we considered the following:

1. Two grasping events are not likely to take place in rapid succession. From the training data, we could infer an estimate of the minimal interval between consecutive grasping events. In our analysis we, therefore, introduced a refractory period by assuming that the time between two successive grasps is always larger than 1.5 seconds. This is a conservative estimate, since the shortest intervals encountered between any two grasps were 3.6, 4.1 and 2.1 seconds for subjects S1, S2 and S3, respectively. Therefore, once an event was detected, we could safely discard potential detection times within the following 1.5 second interval.
2. If detections are delivered *t_advance_* before the time they are required (determined by the *delay*), the output of the detection algorithm can be delayed over this time. This time interval can be used to wait for even more likely detections i.e., data frames yielding a higher posterior probability for an event. If a higher posterior probability is found within this interval *t_advance_*, the detection can be shifted forward accordingly. This rule can be applied recursively until no higher values are found within *t_advance_*. We therefore always based detections on signals, picked from times at 3 evaluation steps before the given *delay* (*t_advance_* = 3/64 s).

By following the reasoning in (2), detection times were advanced towards a maximum in posterior probability. This drastically reduced – but, on average, not completely eliminated – a bias of detections being made too early. Applying a refractory period of 1.5 s, for reasons described in (1), ensured that only one detection was delivered during a period of likely detection.

### Evaluation of Detections

#### Sensitivity, specificity and precision measures

The quality of event detection is determined by the numbers of true positives *N_TP_* – events that were correctly detected – and the numbers of false positives *N_FP_* – detections that occurred despite the absence of an event. The number of false negatives – events missed by the detector – can simply be calculated as the difference between the total number of events *N_events_* and the number of true positives *N_TP_*. To compare these measures across different data sets, we defined the true positive ratio (*TPR*) as the fraction of true positives among all real events *N_events_*. This measure reflects the sensitivity of detection. Additionally, we defined a measure of specificity, the false positive ratio (*FPR*), as the fraction of false positives among all detections *N_det_*. This should not be confused with the false positive rate, which gives an estimate of how many false responses should be expected per unit time. Both measures, *TPR* and *FPR*, are bounded between 0 and 1 and have previously been used in this form to evaluate movement detections [4].
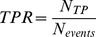


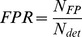



To decide, whether an event was correctly detected, a certain tolerance *τ* of how much the time of the detection was allowed to deviate from the correct time, has to be defined. TPR and FPR are therefore a function of this tolerance *τ*. Fixing *τ* to a defined interval, thus, introduces an implicit measure of temporal precision.

In addition to the above analysis, using a pre-specified tolerance, we determined the temporal error of (potentially) correct detections, by considering for each single event the respective closest detection and measuring its temporal distance to the event. If more than one event referenced one specific detection, only the distance to the closest event was considered (leaving some events undetected). Thereby, we obtained an overview over achievable precision. We summarized this temporal precision into a single variable, termed temporal deviation (*TD*) by means of the root mean squared error:
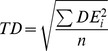



Here, *DE_i_* denotes the detection error, defined as the difference between the time of the *i* ^th^ detection and that of the corresponding event. If the average detection error were zero, *TD* would be identical to the standard deviation, but is larger in the presence of a systematic temporal bias of detections.

#### Baseline detection performance: random predictor

We contrasted the yielded detection accuracy against the null hypothesis that no specific information was extracted from ECoG recordings. To this end we compared results to a random predictor that does not take any information from neural data. We considered a renewal process that triggers detections at a rate equal to that of the true events in the original data and with a minimal inter-occurrence interval of 1.5 s. This provides a suitable comparison to our detection algorithm, because both, the estimate of the rate of events (prior probability) and the minimal inter-event time (refractory period), are part of our detection scheme and independent of neural recordings. *TPR* and *FPR*, assuming tolerances smaller than half the refractory period (0.75 s), can easily be calculated for this process. For decisions on ‘detection’ or ‘no detection’, made in steps of Δ*t*, with a tolerance *τ* in a session of length T containing *N_events_* events, we obtain:
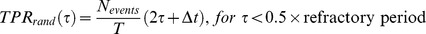


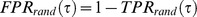



Thus, *TPR* and *FPR*, expected from a random detector, are linear functions of *τ* (for non-overlapping tolerance windows around events, which in our case holds for *τ* <0.75 s) and depends on the temporal density of events. For the derivation, we refer to Appendix S1.

For a detection method based on neuronal recordings to be useful, it should, at the very least, be superior to a random predictor with regard to both, *TPR* and *FPR*.

For a sensitive but unspecific detection method, that is, one that detects multiple time points, besides the desired events, a large number of false detections and hence *FPR* >1-*TPR* would be predicted. On the other hand, a method with high specificity but low sensitivity i.e., one that detects the right kinds of events but only part of them, would be marked by *FPR* <1-*TPR*. On the extreme end of those cases are trivial predictors, producing either a *TPR* of 1 or an *FPR* of 0, by triggering a detection either in every single time bin (*TPR* = 1) or never (*FPR* = 0). Trivial predictors of this kind would, at the same time, produce a *FPR* of almost 1 (for the over-sensitive method, with *TPR* = 1) or a *TPR* of 0 (for the insensitive method, with *FPR* = 0). Superiority over these trivial methods should be documented by a combination of favourable *TPR* and *FPR* with *FPR ≈* 1-*TPR*, as would be predicted if all events were detected, but with a random temporal jitter. Further criteria may be imposed, for instance that the number of true positives should exceed that of false positives. Besides these considerations, the ultimate requirements for *TPR* and *FPR* will depend on the intended application.

#### Cross-validation

To test detection performance, we applied a ten-fold cross-validation to the available data. Recordings from each subject were sub-divided into ten periods of equal length. Nine of these periods were combined to form the training set, providing the basis for estimating the detector parameters. The remaining subset was then used as a test set, to determine the detection performance, based on the trained model. This procedure was repeated for each combination, with one part being used as a test set and the remaining nine parts being combined into a training set. Thus, detection was run once on the complete stretch of available data, with test and training sets being mutually exclusive at any given time.

To choose the regularization parameter 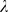 (see section ‘Event detection’) during training, we tested the detection performance as a function of 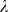 by evaluating a selection of 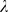-values in a separate ten-fold cross-validation on the current training set (but excluding the test set). The value of 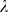 which yielded the best discrimination between events and non-events was then used to retrain the RLDA on the complete training set.

## Results

### Detection from Time-frequency Amplitudes

**Figure 4:**
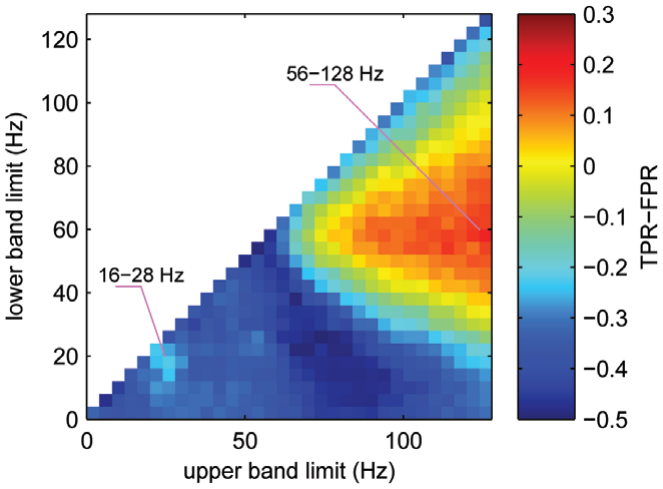
Detection from band-limited ECoG amplitudes. Detection accuracy, obtained using amplitudes from different frequency bands of the ECoG. Every continuous frequency band between 0 and 128 Hz, constructed by averaging over normalized amplitudes of successive frequency bands of 4 Hz width, was used as input to the detection algorithm. The figure shows colour-coded average values of *TPR-FPR*, for frequency bands stretching from a lower limit (vertical axis) to an upper limit (horizontal axis). *TPR-FPR* values were averaged over delays of −0.25 s, 0 s and +0.25 s and tolerances of 125 ms, 250 ms, 375 ms, …, 750 ms as well as over all three subjects (pictures for individual delays and tolerances, as well as individual subjects provided as supporting information, figures S1 and S2). Detections from each frequency band were based on a history of one second, sampled every 62.5 ms (16 times per second), recorded from all available hand-arm motor channels in each subject. Pink lines point to two local maxima in detection performance, representing frequency bands that were used for further analysis.

Commonly used signal components for decoding of movement parameters include amplitudes or power in frequency bands that are modulated during movement or movement planning. To investigate which frequency bands were informative for the detection of grasping event times, we employed our detection strategy (cf. Methods) on the basis of any possible contiguous frequency band between 0 and 128 Hz that could be constructed by averaging over normalized amplitudes in consecutive bands of 4 Hz width. This was repeated for several possible *delays* and the performance was evaluated in each case for a number of different tolerances. Figure 4 shows detection performance as a function of the lower and upper frequency bounds, averaged over different *delays* (−250 ms, 0 and +250 ms) and tolerances *τ* (125 ms, 250 ms, 375 ms, …, 750 ms), and averaged across subjects. The length of the signal history in this evaluation was fixed to 1.25 seconds. As a measure of performance we used *TPR*(*τ*)*-FPR*(*τ*), which weighs sensitivity against specificity and has previously been used in related studies in this [4] or similar form [6], [20]. Figure 4 shows average *TPR-FPR* values for different frequency bands, sorted for lower and upper frequency limits on the vertical and horizontal axes, respectively.

We found that amplitude values recorded from hand-arm motor channels allowed for best performance in a broad high-gamma band, and a bit weaker but still notable performance in a frequency band spanning the beta-range (cf. figure 4). We assured that these findings did not vary substantially over *delays*, tolerances or subjects (figures S1 and S2). Local maxima in performance were found for a 56–128 Hz band in the high-gamma range and for a 16–28 Hz band in the beta range (locations indicated in figure 4). For a closer inspection of the detection performance, we restricted ourselves in the sequel to amplitudes from these two bands (along with the LFC), which for brevity, we termed β for the 16–28 Hz band and γ for the 56–128 Hz band.

### LFC and Combination with Frequency Band Modulations

**Figure 5:**
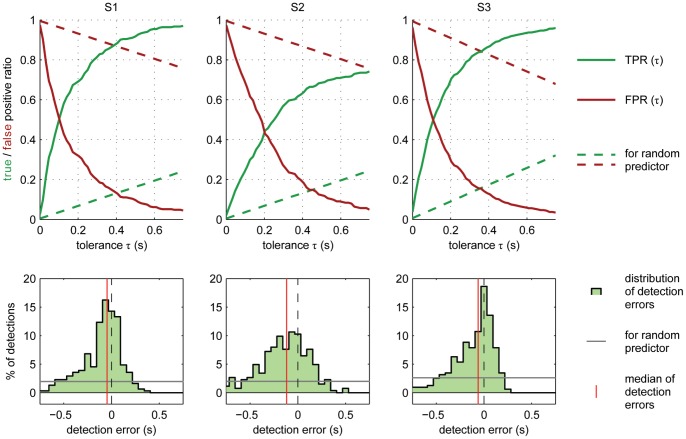
Sensitivity, specificity and temporal precision of detection. Top row: true positive ratio (TPR, green solid trace) and false positive ratio (*FPR*, red solid trace) as a function of tolerance (required temporal precision) for each of the three subjects (different panels). Dashed lines show *TPR* and *FPR* (green and red, respectively) of a random predictor (see Methods, section ‘Evaluation of detections’). Bottom row: distributions of temporal errors of detections. Red vertical lines indicate median temporal error; grey horizontal lines indicate the (flat) distribution for the random predictor (in the same binning). Detection results, summarized here, were calculated for a delay of 0 s, using 1.25 s of signal history (sampled every 62.5 ms within this period) from LFC, 16–28 Hz (β) amplitudes and 56–128 Hz (γ) amplitudes from all hand-arm motor electrodes. *TPR* indicates sensitivity of detections, whereas *FPR* quantifies specificity (with high *FPR* meaning unspecific detections).

Detections from the LFC were generally more accurate than those from either β- or γ-band. A combination of the LFC, together with either β- or γ-amplitudes or both could further improve the accuracy of prediction. Figure 5 shows the performance for a combination of all three signal components, using a signal history of 1.25 s and a *delay* of 0 s. The curves for *TPR*(*τ*) and *FPR*(*τ*) (figure 5, top row) show almost symmetric behaviour with respect to a horizontal line at 0.5 on the *TPR* and *FPR* axis, since an increase in tolerance will classify additional detections as true positives, formerly interpreted as false positives. This indicates that the difference *TPR-FPR* might be a valid summary of these two parameters, as long as the number of real events does not differ too much from the number of detected events, by which the numbers of true and false positives, respectively, were normalized. Also, note that the detection performance was always better than that of a random process, denoted by dashed lines in the top panels of figure 5. In order to reach high levels of correct detections, higher tolerances had to be allowed (see table 3). For S2, more than for S1 and S3, detections lacked in temporal precision, as illustrated by its broader distribution of detection errors in figure 5 (bottom row). Detection errors in S2 were also more biased towards a negative temporal error (predicting too early), and less so in S1 and S3. An overview of numerical values of *TPR*, *FPR*, and *FP-rate* (false positive rate) for this detection scheme is given in table 3. It should be noted that false positive rates given in table 3 (false detections per minute) were calculated for time periods consisting of a sequence of grasping trials and short resting periods in between and warrant no statement about false positive rates of grasp detection during periods of different behaviour.

**Table 3:** Detection accuracy using combined LFC, 16–28 Hz and 56–128 Hz amplitudes.

	*τ* = 0.25 s			*τ* = 0.5 s			*τ* = 0.75 s			*N_det_*	detection errors	
	*TPR*	*FPR*	*FP-rate* (min^−1^)	*TPR*	*FPR*	*FP-rate* (min^−1^)	*TPR*	*FPR*	*FP-rate* (min^−1^)		*Bias* (ms)	*TD* (ms)
**S1**	0.75	0.26	2.5	0.92	0.10	0.9	0.97	0.05	0.4	309	−47	265
**S2**	0.50	0.36	2.7	0.69	0.12	0.9	0.74	0.05	0.4	264	−117	362
**S3**	0.75	0.25	3.1	0.91	0.08	1.0	0.96	0.03	0.4	318	−63	271

True positive ratio (TPR), false positive ratio (FPR) and false positive rate (FP-rate) are given for three different tolerance values τ, 1.5 s of signal history and a delay of 0 s (cf. figure 5). bias: median detection error over all potentially correct detections (negative values indicate detections are positioned before real events). N_det_: number of detections; TD: temporal deviation, measured as the root mean squared error of (potentially) correct detections (see section ‘Evaluation of detections’).

**Figure 6:**
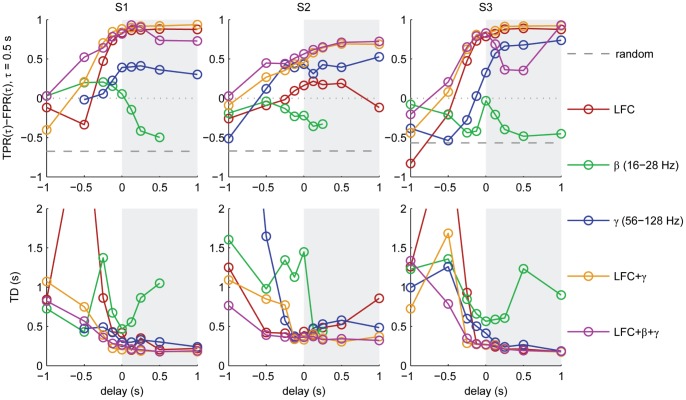
Detection accuracy as a function of delay for different signal components. Detections were inferred from a signal history of 1.25 s at and before the indicated delay of either LFC, β-amplitudes, γ-amplitudes or a combination of these, as indicated in the colour code. Accuracy is represented by *TPR-FPR* for a tolerance of 0.5 s (top row) and the temporal deviation (*TD*) of detections (bottom row) as a measure of overall temporal imprecision. The left half of each plot (white background) comprises delays <0, indicating prediction i.e., event detections ahead of time. In the case of missing data points (β-amplitudes, S1/S2), no events were detected.

A comparison of the performance of different signal components (figure 6) revealed that the lower detection accuracy for S2 can largely be attributed to the low performance obtained, when using the LFC, which for the other subjects provided most of the information.

### Dependence of Performance on Detection Parameters

Early predictions – detections with negative *delays* – could allow BMI applications time for preparation of an appropriate action. This time could be used, for instance, to prepare the grasping movement of a prosthesis. However, in applications in which timing is not crucial, or which have a high tolerance in terms of temporal precision, detection at positive *delays* i.e., detection after the actual event, could be allowed, if this improves overall performance. To test this idea, we carried out detection over a wide range of possible *delays*, from −1 s to +1 s. Dependency of the detection accuracy on *delay* in terms of the *TPR-FPR* measure for an intermediate tolerance of 0.5 s and in terms of *TD* is shown in figure 6. In the prediction phase (*delay* <0, white area), accuracy increased strongly when *delays* got closer to zero. Delayed detection (*delay* >0, grey area), only slightly increased the detection accuracy further.

Detection performance showed only a weak dependence on the duration of the signal history gathered in the feature vector (figure S3). Here, we only presented examples with a fixed history length of 1.25 s.

## Discussion

Using the presented detection method, we were able to detect grasping of a cup against a background of other movement events, such as start of reach, cup release and end of reach. Event detection, using ECoG recordings from motor cortex, worked without information on the temporal structure of the trials. This demonstrates that our method, based on linear discriminant analysis, works reliably and is specific to one class of events, even though the grasps themselves varied in the applied grasp type, weight of the lifted object and position in the workspace. Grasping events could also be predicted 125–250 ms before their occurrence, without substantial loss in accuracy, allowing for an early preparation signal in potential future applications.

### Detection Algorithm

Distinguishing between two classes, with one class representing a specific event and the other class everything else, might be taken to imply that distributions of neural features of both classes are probably quite different and therefore not well described by a common class covariance, as it is assumed and used in linear discriminant analysis. Therefore, we tested our detection algorithm also using RDA (regularized discriminant analysis [19]) which, unlike RLDA, allows for class specific covariances. This, however, did not yield better predictions than RLDA (data not shown), as long as samples, very close to events were excluded from the training set of non-events (see Methods, section ‘Event detection’). For this reason, we here only presented detailed results of the RLDA approach.

### Precision

While predictions were quite reliable at sub-second precision, the observed temporal deviations might still be too large for applications of very precisely timed movement control, such as catching a ball or interaction with fast moving objects. An explanation for these temporal deviations may be found in the time course of the posterior probability, which was the basis for our event detection: within intervals of several hundred milliseconds duration around grasping events, the posterior probability for an event was higher than the used threshold of 0.95. While these peaks in the posterior probability featured a steeply rising flank, the maximum was often located on a plateau-like episode (cf. figure 3, inset). Simply raising the detection threshold further than 0.95 might slightly narrow this interval, but at the expense of decreased sensitivity, that is, the danger of more events being missed by the detection. Optimizing the final detection time, by climbing the gradient in the posterior towards a maximum brought detections closer to the correct event times as exemplified in figure 3 (grey arrow), but still left considerable temporal ambiguity.

The temporal profile of the posterior probability can most likely be attributed to autocorrelations in the signal components used for detection. Using signal components with more transient event-specific potentials could potentially improve the temporal precision of detections. But even if these existed in the motor-cortical ECoG, they might be difficult to detect since events of the training set, marked on the basis of movement behaviour, had only limited temporal precision and, therefore, cannot reveal transient signals which are locked to the event on very short time-scales. This, in fact, might be a general difficulty for self-paced movements and smooth transitions between movement components, like reach and grasp.

### Specificity

For all three subjects, *FPR*s converged towards a value close to zero, for large tolerances (Fig. 5). This suggests that most false detections were due to a temporal scattering around the actual event times, indicating limited precision, rather than unspecific triggering of detections. This is all the more remarkable as the analysed data not only included two kinds of grasps and periods of rest (see Table 2), but also a large variety of movement components, like reaching to and from different positions and carrying cups of two different weights. However, any analysis of specificity within the limits of such an experiment will not allow for a very general statement of how many false detections are to be expected during every-day activities, as this would require monitoring of movement and ECoG over a much broader range of behaviour.

### Comparison to Previous Studies on Movement Detection

So far, only few studies reported on the detection of movement events in time from neural signals. Rather than looking for specific events within a continuous movement, most of these previous studies were concerned with detection of movement onset of reaching movements (from LFP: [3]) or short, simple movements (from ECoG [4]; from EEG [5], [6]) or other movement related states (e.g., detection of a planning phase from spiking activity in monkey pre-motor cortex [21]; detection of periods of event-related desynchronization and synchronization from human EEG [20]). In this respect, our study extends and complements previous investigations.

Moreover, earlier studies did not emphasize the aspect of temporal acuity, even though this aspect substantially influences sensitivity and specificity of detection and is crucial to determine the range of possible applications.

We specifically targeted hand and arm areas of the human motor cortex, a site likely to be targeted in future BMI applications. This not only reduces the impact of post-central sensory sources, but also allows for a more specific statement about potential capabilities of epi-cortical BMIs than permitted by previous studies based on EEG [5], [6].

### Conclusion

Augmented with additional classification of the applied grasp type [7], our findings introduce a possible approach for the development of an ECoG-based brain-machine interface for grasping. Moreover, our detection methods are of interest for detecting other events (e.g. movement onset, error signals) from neuronal data.

## Supporting Information

Figure S1 Detection accuracy, using different frequency bands (see manuscript, figure 4), for different delays and tolerances (*τ*). Average over 3 subjects.
(EPS)

Figure S2 Detection accuracy, using different frequency bands (see manuscript, figure 4), for single subjects. Average over *delays* and tolerances, displayed in figure S1. White spaces below the diagonal appear, if no detections were made for the according frequency band.
(EPS)

Figure S3 Detection accuracy as a function of signal history. 0.5 to 1.5 s of signal history before the time given by the *delay* (here: *delay = *0) included into the feature space. Different signal components or combination of components are marked by colour (see legend). Missing data points (β) indicate that no detections were made.
(EPS)

Appendix S1 Calculation of TPR and FPR for a random process (cf. section ‘Baseline detection performance: random predictor’).
(DOCX)
