# Supplementary material for: Grasp Detection from Human ECoG during Natural Reach-to-Grasp Movements
Source: PLoS One. 2013 Jan 24;8(1):e54658. doi: 10.1371/journal.pone.0054658 (PMC3554656; doi:10.1371/journal.pone.0054658)
Supplement: Appendix S1 — Calculation of TPR and FPR for a random process (cf. section ‘Baseline detection performance: random predictor’). (DOCX) [file pone.0054658.s004.docx]

# Appendix S1

### Calculation of TPR and FPR for a random process (cf. section ‘Baseline detection performance: random predictor’)

In the random detection process, detections are generated randomly with equal probability in each “observation” time bin of width Δ*t*, unless a (random) detection was made less than one refractory period ago, in which case the probability of a new detection is zero. For this random process, the average probability *p_det_* of observing a detection in a time bin is

$p_{det}=\frac{N_{det}}{N_{bins}}$ (A.1)

with *N_det_* being the total number of detections and *N_bins_* the total number of time bins in the observation interval, calculated as

$N_{bins}=\frac{T}{\Delta t}$ (A.2)

where *T* is the total length of the session.

If real events are sampled at the same time resolution Δ*t*, sensible values for the tolerance *τ* are multiples of Δ*t* ($\tau\in\left\{\text{0,}\Delta t,2\Delta t,3\Delta t,\ldots\right\}$). Thus, the length of a tolerance window is given as

$T_{\tau}=2\tau+\Delta t$ (A.3)

harbouring *n_τ_* bins:

$n_{\tau}=\frac{T_{\tau}}{\Delta t}$ (A.4)

We considered a tolerance window around each real event. These tolerance windows did not overlap as long as *T_τ_* ≤ refractory period i.e., *τ* < 0.5 × refractory period, since the refractory period was chosen smaller than the smallest interval between any two events (section ‘Evaluation of detections’). Therefore, the expected number of detections that fell in time bins within any of those tolerance windows can be calculated as

$N_{TP}\left(\tau\right)=p_{det}\cdot n_{\tau}\cdot N_{events}$, (A.5)

which is the number of true positives, since these detections were all close enough to real events.

Therefore, we can summarize (A.1)-(A.5) as

$N_{TP}\left(\tau\right)=\frac{N_{det}\cdot\Delta t}{T}\cdot\frac{\left(2\tau+\Delta t\right)}{\Delta t}\cdot N_{events}=\frac{N_{det}\cdot N_{events}}{T}\cdot\left(2\tau+\Delta t\right)$ (A.6)

With

$TPR\left(\tau\right)=\frac{N_{TP}\left(\tau\right)}{N_{events}}$ (A.7)

*N_TP_*(*τ*) can be substituted by (A.6). Assuming that $N_{det}=N_{events}$, *TPR*(*τ*) can be calculated as

$TPR\left(\tau\right)=\frac{N_{events}}{T}\cdot\left(2\tau+\Delta t\right)$. (A.8)

All remaining detections outside the mentioned tolerance windows must be false detections, hence

$FPR\left(\tau\right)=1-TPR\left(\tau\right)$. (A.9)
